# Case-Control Study on the Role of Enterotoxigenic *Bacteroides fragilis* as a Cause of Diarrhea among Children in Kolkata, India

**DOI:** 10.1371/journal.pone.0060622

**Published:** 2013-04-05

**Authors:** Dharanidharan Ramamurthy, Gururaja P. Pazhani, Anirban Sarkar, Ranjan K. Nandy, Krishnan Rajendran, Dipika Sur, Bamkesh Manna, Thandavarayan Ramamurthy

**Affiliations:** National Institute of Cholera and Enteric Diseases, Kolkata, India; University of Illinois at Chicago, United States of America

## Abstract

A total of 874 fecal specimens (446 diarrheal cases and 428 controls) from diarrheal children admitted in the Infectious Diseases Hospital, Kolkata and age and sex matched asymptomatic subjects from an urban community were assessed for the prevalence of enterotoxigenic *Bacteroides fragilis* (ETBF). Isolates of *B. fragilis* were tested for the presence of enterotoxin gene (*bft)* by PCR. The detection rate of ETBF was 7.2% (63 of 874 specimens) that prevailed equally in diarrheal cases and controls (7.2% each; 32 of 446 cases and 31 of 428 controls). Male children up to one year age group was significantly (p<0.05) associated with ETBF infection as compared to children > 2 years of age in cases and controls. In 25 ETBF isolates, the *bft* gene was genotyped using PCR-RFLP and only two alleles were identified with prevalence rate of 40% and 60% for *bft-1* and *bft-3*, respectively. All the ETBF isolates were susceptible for chloramphenicol and imipenem but resistant to clindamycin (48%), moxifloxacin (44%) and metronidazole (32%). Resistance of ETBF to moxifloxacin (44%) and metronidazole is an emerging trend. Pulsed-field gel electrophoresis (PFGE) revealed that majority of the ETBF isolates are genetically diverse. In the dendrogram analysis, two clusters were identified, one with ETBF resistant to 5–8 antimicrobials and the other cluster with metronidazole and moxifloxacin susceptible isolates from diarrheal cases. To our knowledge, this is the first detailed report on ETBF from India indicating its clinical importance and molecular characteristics.

## Introduction

The *Bacteroides* species are a group of Gram-negative anaerobes, which generally represent as a major constituent of the human gut microbiota. Although *Bacteroides* species play an important role in mediating mucosal and systemic immunity, this group sometimes cause opportunistic infections. *Bacteroides fragilis* is the only known species of the *Bacteroides* that can cause diarrhea and frequently isolated from abscesses, soft tissue infections and bacteremia [1]. *B. fragilis* does not have other known niches except the gut of mammals [2]. A study conducted in southern India showed that *B. fragilis* was frequently detected in humans with and without diarrhea [3]. In India, this pathogen has been identified in different clinical specimens and also in healthy persons but its virulence factors are not confirmed [4]. *B. fragilis* is categorized into two subgroups: non-enterotoxigenic *B. fragilis* (NTBF) and enterotoxigenic *B. fragilis* (ETBF). In developing counties, ETBF is an emerging pathogen associated with diarrhea in children and travelers [5]–[7]. In children, ETBF is associated with secretory diarrhea with mild severity and hence much attention has not been paid to this pathogen. The other syndromes of ETBF associated infection include extraintestianl infections, abdominal pain, tenesmus, inflammatory diarrhea, antibiotic associated diarrhea and chronic inflammation that lead to colon cancer [8]–[11].

ETBF produces a specific virulence factor known as fragilysin, which is a heat-labile enterotoxin responsible for mucosal inflammation. Based on the sequence variation in the *B. fragilis* enterotoxin encoding gene *bft*, three subtypes namely *bft-1*, *bft-2*, and *bft-3* have been identified and these are predominantly found in specific geographical locations [10]. Several methods have been reported for the diagnosis of ETBF including conventional culture technique, cell culture assay, enzyme immunoassays, immunomagnetic separation followed by PCR (IM-PCR), and nested PCR [10]. Among these methods, nested PCR has been considered as a most simple and sensitive method [10], [11]. As this pathogen is associated with a wide variety of infections, information regarding its prevalence and characterization are important for the successful clinical management.

In India, only few studies have been made to detect ETBF associated with diarrhea or among non-diarrhea patients [3], [4], [6], [12]. This study was undertaken to detect the prevalence of ETBF among children with diarrhea admitted in the Infectious Diseases Hospital (IDH), Kolkata and without diarrhea from an urban community. In addition, phenotypic and genotypic characteristics of the ETBF were also investigated.

## Materials and Methods

### Study populations and sample collection

From February to August 2012, 874 fecal specimens collected from children below five years of age were processed. Of these, 446 fecal specimens were obtained from diarrheal children treated in the IDH and 428 samples of age and sex matched asymptomatic controls from an urban community. Before the initiation of antimicrobial therapy, the stool specimens were collected from these children. The children admitted in the hospital were treated with intravenous fluid (IVF) or oral rehydration solution (ORS) depends on the nature of dehydration and oral ciprofloxacin (6–10 mg per Kg of the body weight) and metronidazole (35–50 mg per Kg) was given in divided doses. After microbial screening, aliquots of the fecal specimens were stored at−80°C for subsequent use.

### Extraction of total nucleic acid from fecal specimens

Fecal specimens (∼100 mg semi solid or 200 µl if liquid) were suspended in nuclease free water (final concentration ∼10%) with equal volume of vortel XF (Miller-Stephenson Chemical Co, Inc, Danbury, CT) and vortexed for 2 min followed by centrifugation at 4500 rpm for 10 min. Two hundred micro liters of the supernatant was used for extraction of the total nucleic acid using an automated system (NucliSens EasyMAG; bioMérieux, Marcy l'Etoile, France).

### Culture and confirmation of ETBF

Stool specimens collected from patients and controls were transported to the laboratory within 2 hrs of collection in a cold chamber maintained at 4°C. The fecal specimens were streaked on to respective selective agars for isolation of vibrios, *Salmonella* spp, *Shigella* spp, *Campylobacter* spp, and *Aeromonas* spp and identified these pathogens as described before [13]. Three different *Escherichia coli* colonies from MacConkey agar were tested for different pathogroups by multiplex PCR [13]. For the isolation of *Bacteroides* species, Bile-Esculin (BBE) agar (Becton, Dickinson and Company, Sparks, MD) plate was used and incubated at 37°C for 48 hrs in an anaerobic jar (BD GasPak EZ anaerobic systems). After incubation, several individual gray, raised circular colonies surrounded by esculin hydrolyses from each specimen was subcultured on Colombia blood agar (CBA) plate (bioMérieux) and incubated anaerobically for 48 hrs. A portion of pure culture from the blood agar was suspended in an anaerobic broth [Luria Broth supplemented with beef extract (0.3%), cysteine HCl (0.04%), glucose (0.1%) sodium hydrogen phosphate (0.4%) and glycerol (15%)] and preserved at −80°C. The remaining portion of the culture was suspended in TE buffer for the confirmation of ETBF by PCR. In addition to culture methods, ELISA was performed to detect rotavirus, adenovirus, and parasites such as *Giardia lamblia*, *Cryptosporidium* spp. and *Entamoeba histolytica* directly from the stool specimens [13]. *Helicobacter pylori* in the stools were detected using a commercial ELISA kit (Amplified IDEIA™ HPStAR, Oxoid, Basingstoke, Hants, UK). The DNA extracted from the stool specimens were used for the detection of Astro virus, Sapo virus, and Noro virus (Genotype I and Genotype II) by reverse transcriptase PCR [13].

### Detection of *B. fragilis* and ETBF by PCR

The pure cultures of *Bacteroides* from CBA plates were suspended in a TE buffer and boiled for 15 min in the water bath, snap cooled on ice and centrifuged at 10, 000 rpm for 5 min. The resulting supernatants were screened for the 16S-rRNA gene-specific for *B. fragilis* group and *bft* by PCR [14], [15]. In addition, the total nucleic acid extracted from the fecal specimens was tested for the presence of *bft* by PCR.

### Genotyping of *bft*

From the 63 ETBF positive stool specimens by PCR, 30 ETBF were isolated by culture method. Due to non-viability of 5 isolates, only 25 isolates harboring *bft* was amplified by PCR and restriction fragment length polymorphism technique (RFLP) was applied to detect the *bft* subtypes [16]. The PCR amplified products were purified using QIAquick PCR purification kit (QIGEN, GmBH, Hilden, Germany) and then digested with *Sau3A*1 (Thermo Scientific Inc., Waltham, MA) according to the manufacturer's recommendations. The digested DNA was separated in a 2% agarose gel, and visualized after ethidium bromide staining.

### Antimicrobial susceptibility testing

ETBF isolates were grown on CBA and suspended in sterile saline and the cell density was determined using a densitometer (bioMérieux), which is equivalent to a 1.0 McFarland standard (∼3×10^8^ CFU/mL). The cell suspension was spread uniformly on the Brucella blood agar (BBA, Oxoid, Basingstoke, UK) supplemented with 5% laked sheep blood, hemin, and vitamin K according to CLSI guidelines [17]. After the inoculation, E-test strips (AB Biodisk-bioMérieux) for each drug (amoxicillin-clavulanic acid, AMC; ampicillin, AMP; ampicillin-sulbactam, SAM; cefoxitin, FOX; chloramphenicol C; ciprofloxacin, CIP; clindamycin, CLI; imipenem, IPM; moxifloxacin, MXF; norfloxacin, NOR; and metronidazole, MTZ) was placed and incubated for 48 hrs at 37°C in an anaerobic atmosphere. Reference strain *B. fragilis* ATCC 25285 was used as control. Resistant and susceptibility of ETBF were estimated according to quality control ranges for *B. fragilis* assigned by the manufacturer's instruction and also using breakpoints information of CLSI and other reports [17]–[19].

### Pulse Field Gel Electrophoresis (PFGE)

PFGE protocol described by Yamasaki et al. [20] was slightly modified and adopted in this study. Briefly, 25 ETBF isolates were anaerobically grown for 14–18 hrs on CBA plates at 37°C. Bacterial cultures were suspended in cell suspension buffer (CSB; 10 mM Tris-HCl (pH 7.2), (20 mM NaCl) (50 mM EDTA [pH 8.0]) using sterile cotton swabs. Cells were harvested by centrifugation, washed and resuspended in CSB and adjusted to an optical density of 1.0 to 1.2 at 610 nm. The cell suspension (500 µl) was mixed with (500 µl) molten low melting agarose (2%) at 50°C. The mixture was carefully dispensed into a sample mold (Bio-Rad, Hercules, CA). After solidification, the plugs were transferred to a 2.0-ml micro centrifuge tubes containing 1.0 ml of cell lysis buffer (1 mg/ml lysozyme, 10 mM Tris-HCl (pH 7.2) 50 mM NaCl, 0.2% sodium deoxycholate and 0.5% of sodium laurylsarcosine) and incubated at 37°C for 3 hrs. After incubation, plugs were washed twice with reagent grade water and treated with 1 ml of proteinase K solution (1 mg/ml proteinase K, 100 mM EDTA (pH 8.0) 0.2% sodium deoxycholate and 1% of sodium laurylsarcosine) at 50°C for overnight. The plugs were washed with washing buffer (10 mM Tris-HCl and 50 mM EDTA [pH 8.0]) two times for one hr, once with phenylmethylsulfonyl fluoride (1 mM) containing wash buffer and twice with diluted wash buffer (0.1X) with agitation at room temperature.

The agarose-embedded ETBF DNA plugs were digested with 50 U of *Not*I enzyme (New England Biolabs Inc., Ipswich, MA) and *Salmonella enterica* serovar Braenderup (H9812) was digested by *Xba*I and its DNA fragments were used as molecular size markers. The digested DNA fragments were resolved with 1% PFGE-grade agarose (SeaKem Gold agarose, Lonza, Rockland, ME) in 0.5X trisborate EDTA buffer at 6 V/cm for 16 hrs at 14°C. Run conditions were generated by the autoalgorithm mode of the CHEF Mapper PFGE system (Bio-Rad) with a size range of 30–600 kb. After electrophoresis, the ethidium bromide (Sigma, St. Louis, MO) stained agarose gel was visualized and the captured images were digitized for computer-aided analysis (Gel Doc system, Bio-Rad). PFGE profiles were analyzed using the BioNumerics version 4.0 software (Applied Maths, Sint Martens Latem, Belgium). The tagged image file formats were normalized by using the universal *Salmonella enterica* serotype Braenderup (H9812) size standard on each gel against the reference in the database. PFGE profiles were matched using the Dice coefficient and unweighted pair group method using arithmetic averages (UPGMA) clustering with a 1.5% band position tolerance window and 1.5% optimization. The clustering of the PFGE patterns and band assignments were verified visually.

### Statistical Analysis

The inferential age groups were evaluated for ETBF positive specimens from under five years children by Multinomial Logistic Regression (MLR)(1,2) analysis using SPSS software (Version 19.0, SPSS Inc., Chicago, Illinois). The age groups were classified into 3 categories: ≤1 year, >1–2 years and >2 years and coded as 1–3, respectively. The relationship between the risk dependent variable and each of the categorical explanatory variables are shown in Table 1. Infection caused by a ETBF was classified in number as ‘1’ for organism present and ‘2’ for its absence. The extreme values of the classified age group was fixed as reference category (>2 yrs).

**Table 1:** Multinomial Logistic Regression Models exploring significant risk age group of ETBF infection.

	Age	B-value	OR 95% CI	*P* value
Cases				
	Up to 1 year	0.944	2.57 (1.07–6.16)	0.034*
	Male	1.83	6.57 (1.08–39.88)	0.041*
	Female	1.33	3.80 (0.64–22.4)	0.141
	1–2 years	0	1.00 (0.35–2.85)	1
	Male	0	1.00 (0.12–8.54)	1
	Female	0	1.00 (0.12–8.28)	1
	>2 years		Reference category	
Controls				
	Up to 1 year	1.041	2.83 (1.12–7.19)	0.028*
	Male	2.06	7.87 (1.1951.97)	0.032*
	Female	1.77	5.91 (0.88–39.43)	0.067
	1–2 years	0.288	1.33 (0.46–3.84)	0.594
	Male	0.71	2.03 (0.24–17.31)	0.516
	Female	0.778	2.18 (0.25–18.71)	0.478
	>2 years		Reference category	

* Statistically significant

### Ethics Statement

Ethical approval was obtained from the National Institute of Cholera and Enteric Diseases Ethics Committee (Ref.C-48/2012-T&E), and parents of the children gave written informed consent.

## Results

### Prevalence of ETBF

A total of 874 fecal specimens were analyzed in this study including 446 from diarrheal children and 428 from controls. The overall detection rate of ETBF was 7.2% (63 of 874) that prevailed evenly in cases and controls (7.2% each; 32 of 446 cases and 31 of 428 controls). ETBF was detected as the sole pathogen in 14 of 32 (44%) cases and 12 of 31(39%) controls. However, these results were not statistically significant. Thirty-five ETBF positive samples were associated with different enteric pathogens and equally found in cases and controls (57% each). Details of co-pathogens associated with ETBF are presented in Table 2.

**Table 2:** Prevalence of ETBF as sole and with other pathogens in children with diarrhea and controls.

Pathogen	Case (n = 446)	Control (n = 428)
ETBF as a sole pathogen	**14**	12
Adenovirus	**1**	**1**
*C. difficile*	**2**	**1**
*Campylobacter jejuni* and Adenovirus	**1**	
*C. jejuni* and EPEC	**1**	
*C. jejuni* and Norovirus GII	**1**	
*C. coli*, Adenoviurs and *Giardia*		**1**
EAEC	**1**	**2**
EAEC and Adenovirus	**1**	
EPEC	**1**	
EPEC, Adeno virus and *Giardia*		**1**
EPEC, *H. pylori*		**1**
Giardia	**3**	**6**
*Giardia* and Adenovirus		**1**
*Giardia, Cryptosporidium*		**1**
*Giardia, H. pylori*	**3**	
*Giardia* and Norovirus GII		**1**
*H. pylori*	**2**	**2**
Norovirus GII		**1**
***Shigella*** ** spp**	**1**	

Comparative analysis revealed that the detection rate of ETBF among the three age groups in both case and control were 18(9.3%), 7 (5.6%), 7 (5.6%) and 17 (10.1%), 8 (6.6%), 6 (4.3%), respectively for up to 1 years, >1–2 years and >2 years. There was no difference between cases and controls in the prevalence of ETBF. However, ETBF detection rate in male children under 1year of age group was significant (p<0.05) in cases as well as controls as compared to>2 years of children (Table 1). By culture method, 30 (47.6%) ETBF isolates (16 and 14 from cases and controls, respectively) were identified from 63 stool DNA-direct PCR positive specimens and all the ETBF isolates were also positive in the species-specific 16S-rRNA PCR. Five ETBF isolates lost their viability during storage.

### ETBF subtypes

In the PCR-RFLP analysis, *bft-1* and *bft-3* alleles of the toxin encoding genes were identified among 25 ETBF isolates. There were no differences in the distribution of these alleles among ETBF isolates from cases and controls (*bft-1* 40% and *bft-3* 60% in cases and controls, respectively).

### Antimicrobial susceptibility testing

Based on the MIC cut-off values, the antimicrobial testing results were categorized as resistant and susceptible. The ETBF isolates were uniformly susceptible to imipenem and chloramphenicol. The resistance frequencies to ampicillin, ampicillin/sulbactum, amoxicillin/potassium clavulanate, cefoxitin, clindamycin, ciprofloxacin, metronidazole, moxifloxacin, and norfloxacin remained 92, 48, 60, 8, 48, 88, 44, 32 and 92%, respectively.

### PFGE

**Figure 1:**
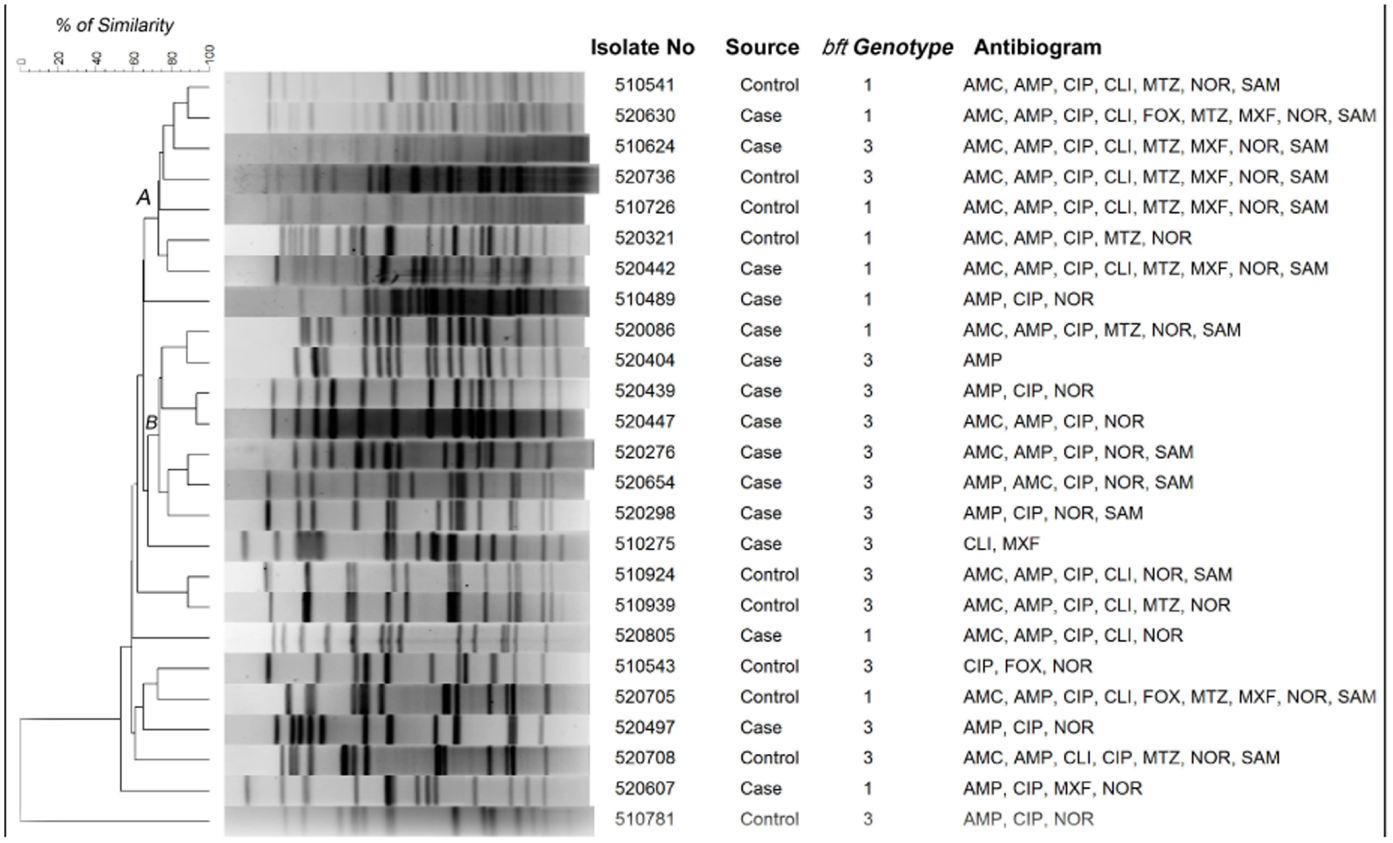
*Not1* restriction patterns of genomic DNA of enterotoxigenic *B. fragilis* isolates. The dendrogram was generated by using UPGAMA method.

PFGE was performed with ETBF isolated from 15 cases and 10 controls. The UPGMA based dendrogram displayed two major clades (A and B) containing 14 isolates with 75% homology (Fig. 1). Except two isolates in the clade B, majority of the ETBF remained genetically heterogeneous and there is no clear demarcation of ETBF isolated from cases and controls. However, majority of ETBF in clade B were isolated from diarrheal cases. In the clade A, of the 7 ETBF, 4 were from controls and resistant to 5–8 antimicrobials including metronidazole and moxifloxacin. ETBF isolates in the clade B were resistant for 1–5 antimicrobials but susceptible for metronidazole and moxifloxacin. In addition, all the isolates in clade B harbored *bft-3* (Fig.1).

## Discussion

In this study, *bft*-PCR assay was performed using the DNA extracted from the stools. The designed primers for *bft* amplification cover all the three toxin genotypes. PCR assay for the detection of ETBF is more useful than culture method as the later needs prolonged anaerobic incubation followed by confirmation of the isolates. Of the 63 PCR positive specimens, culture results yielded 47.6% positivity for ETBF, with an overall isolation rate of 3.4%. In a previous study based on culture and *bft*-PCR from Goa, India showed that the detection rate of ETBF among the travelers with diarrhea was 13% [6]. In a study conducted in Kolkata by bacterial culture and toxin assay using tissue culture revealed that isolation rate of ETBF among acute diarrheal cases was 2.6% [12]. Similar to our results, low prevalence of ETBF was documented in Bangladesh (3–4%) and Brazil (2%) [21], [22]. In the HT29/C1 cell assay and the PCR based detection method showed that the association of ETBF is case and controls were not significant (Table 3).

**Table 3:** Prevalence of enterotoxigenic *Bacteroides fragilis* in diarrheal cases and controls in different studies.

Place	Prevalence of ETBE (%)			Detection method	Remark	*bft* genotype (%)	Reference	
	Case	Control				Case	Control	
Bangladesh	22 (6.1) n = 358	5 (1.2) n = 425		HT29/C1 assay	p = 0.0001	ND	ND	[7]
Apache and Bangladesh	44 (4.4) n = 991	18 (3.1) n = 581		HT29/C1 assay	NS	ND	ND	[48]
India	6 (2.6) n = 226	3 (1.7) n = 172		HT29/C1 assay	NS	ND	ND	[12]
Italy	14 (21.5) n = 65	9 (6.9) n = 129		HT29Cl assay	NS	ND	ND	[24]
Bangladesh	28 (3.5) n = 814	12 (1.5) n = 814		HT29/C1 assay	p = 0.01	ND	ND	[21]
Sweden	195 (26.8) n = 728	24 (12.4) n = 194		HT29/C1 assay	NS	ND	ND	[28]
Brazil	2 (2.1) n = 96	0 n = 74		HT29/C1 assay	NS	ND	ND	[22]
Bangladesh	40 (2.3) n = 1750	15 (0.3) n = 5679		HT29/C1 assay	p = 0.001	ND	ND	[30]
Vietnam	43 (7.3) n = 587	6 (2.4)n = 249		Immuno and PCR	p = 0.01	*Bft-1* (67.4), *Bft-2* (18.6) *Bft-3* (16.0)	*Bft-2* (83.0), *Bft-2* (17.0)	[27]
Turkey	13 (11.0) n = 117	8 (7.8)n = 102		PCR	p = 0.05	ND	ND	[25]
Turkey	28 (38.0) n = 73	7 (12.0) n = 59		PCR	p = 0.009	ND	ND	[34]*
Turkey	29 (15.0) n = 200	27 (14.0) n = 200		PCR	NS	*Bft-1* (82.7), *Bft-2* (17.3)	*Bft-1* (88.9), *Bft-2* (11.1)	[23]
Brazil	9 (8.2) n = 110		7 (4.7) n = 150	Real time PCR	NS	*Bft-1* (8.2), *Bft-3* (0.9)	*Bft-1* (4.7)	[46]

Abbreviations: ND, not done; NS, not significant. * Study with colorectal cancer patients

In almost half the number of PCR-positive stools, we could not isolate ETBF, though we tested several typical *B. fragilis* colonies from each specimen. The recovery rate of *B. fragilis* would have been better if we used strict anaerobic conditions at the time of stool collection, transport and during processing. However, with the use of DNA based PCR assay, we could detect the ETBF almost two times more (7.2%) than the culture method. For the detection of ETBF, molecular based detection methods are very useful as the assays are sensitive, rapid and easier to perform. A real-time PCR approach may also be helpful for the rapid diagnosis of ETBF.

This study is the first of its kind as we examined the ETBF burden among young children in India. The prevalence rate of ETBF among diarrheal patients and asymptomatic controls from different countries are shown in Table 3. In accordance with studies conducted in Turkey and Italy, the prevalence of ETBF in Kolkata was almost equal in children with diarrhea and controls [23], [24]. However, reports from Vietnam, Turkey, Apache Indians in USA and Bangladesh, showed that the prevalence of ETBF was significantly high in cases and controls [25]–[27]. The highest prevalence of ETBF has been documented in countries such as Turkey (25%), Sweden (23%), Italy (17%), and in apache region of Arizona-USA (12%).

Overall, the prevalence of ETBF was the same in both cases and controls. However, considering the sole infection status, ETBF was comparatively identified more in cases (44%; 14/32) than in controls (39%; 12/31). In the investigations carried out in Bangladesh, Sweden, Turkey, Japan and Nicaragua, ETBF was detected as the only pathogen from 39 to 88% of the diarrheal cases [9], [25], [28], [29]. These findings support the view that there must be specific geographical difference in the prevalence of ETBF. In addition, findings from several countries show that ETBF significantly high in children with older age group [7], [9], [30]. Our findings show that ETBF was more frequently found in children less than 1 year age group (Table 1).

Polymicrobial etiology in diarrheal cases is a common trend in many endemic regions [31], [32]. We observed that the co-infection rate of ETBF with other pathogens was 4% in children with diarrhea, which is almost similar to the findings from Vietnam [27] or with higher age group patients from Bangladesh and Turkey [7], [23]. The significantly associated pathogens found with ETBF include enteropathogenic *E. coli* (EPEC), *Shigella* spp, *Campylobacter* spp., *Salmonella* spp., *Clostridium difficile, Entameoeba histolytica*, C*ryptosporidium*, *Giardia* spp. Rota virus and Adeno virus [7], [25], [27], [28]. Although we screened for all these pathogens, we found no significant association between ETBF and other pathogens.

Three different genotypes of *bft* have been documented in the ETBF and detection of these genetic signatures is useful in assessing the severity of the infection. Although the BFT has similar biological activity, their toxicity seems to differ based on its genotype. The purified BFT-2 elucidated higher biological activity than the other two genotypes [9], [10]. In addition, the *bft-2* allele harboring ETBF colonize well in the intestines of children than in adults [33] and exhibit antibiotic associated diarrhea [9]. In this study, majority of the ETBF isolates harbored the *bft-3* allele than *bft-1* and none had the *bft-2* allele. ETBF harboring the *bft-1* allele has been reported from many countries (Table 3) [9], [23], [34]. In Turkey, in addition to *bft-1*, *bft-2* allele was also identified in ETBF from diarrheal children and adults [23]. In Japan and Korea, prevalence of ETBF harboring *bft-*3 was reported in septicemia and diarrheal cases [9], [16] but this allele is rarely found in European countries [10], [33], [35]. We identified *bft-*3 predominantly in diarrheal cases and controls in Kolkata and perhaps this is the first report on the prevalence of *bft-3* in Southeast Asia region.

Although ETBF causes self-limiting diarrhea, antimicrobial therapy is recommended to reduce the possibility of imminent extraintestinal complications. Despite antibiotic therapy, intestinal inflammation caused by ETBF may persist for about 3 weeks [15]. Several antimicrobial susceptibility studies have been documented with clinically isolated *B. fragilis* [36]–[38] but only few reports exist on ETBF [34], [39], [40]. Moxifloxacin alone or in combination with metronidazole is advocated for the empirical treatment of infections caused by Gram-negative anaerobes [41]. In addition, cefoxitin, clindamycin, and carbapenems are recommended for anaerobic infections. Recently, acquisition of resistance by *B. fragilis* to many of these antimicrobials has been documented [42]. To generate basic information on resistance nature of ETBF, we have used E-test method in this study, as this assay has been adopted for many anaerobes [19], [43]. We found that the ETBF are susceptible for chloramphenicol, and imipenem, but resistant to moxifloxacin and clindamycin. In addition, only 8 and 44% of the isolates are resistant to cefoxitin and metronidazole, respectively. Studies from in Brazil and Poland have documented that majority of the ETBF produced beta-lactamase, but susceptible for amoxicillin/clavulanic acid, imipenem and metronidazole [34], [44]. Rarely, some of the *B. fragilis* isolates from human stools were resistant for clindamycin and cefoxitin [39].

In the PFGE, the metronidazole and moxifloxacin susceptible and resistant ETBF isolates are clustered into two distinct groups. Overall, the PFGE results with ETBF isolates showed that they are genetically distinct. This trend seems to be common in many countries [39], [45]. Though we have identified many ETBF harboring *bft-1* and *bft-3* in this study, they are genetically different as evidenced from the PFGE. It is well known that in some bacterial species such as *Campylobacter jejuni*, diarrheagenic *E. coli* etc., the genetic constitution is largely diverse and hence the clonality of isolates in any given area may vary extensively.

### Conclusion

This study highlights the prevalence of ETBF in children without any significant association with diarrheal cases or in controls. However, ETBF was significantly detected in male children younger than one year of age group compared to>2 years group. Overall, ETBF was predominantly detected as a co-pathogen along with enteric parasites and viruses. The *bft-3* genotype was mostly seen than *bft-1*, without any specific age groups or the specimen category. Antimicrobial susceptibility results showed that all the ETBF isolates were susceptible to chloramphenicol, imipenem. Resistance of ETBF for clindamycin, moxifloxacin and metronidazole seems to be an emerging trend. Majority of the isolates are genetically heterogeneous as detected in the PFGE. More controlled long-term studies are required to prove the role of ETBF as an etiological agent for diarrhea.
